# School sociodemographic characteristics and obesity in schoolchildren: does the obesity definition matter?

**DOI:** 10.1186/s12889-018-5246-7

**Published:** 2018-03-09

**Authors:** Silvia Bel-Serrat, Mirjam M. Heinen, John Mehegan, Sarah O’Brien, Nazih Eldin, Celine M. Murrin, Cecily C. Kelleher

**Affiliations:** 1 National Nutrition Surveillance Centre, School of Public Health, Physiotherapy and Sports Science, University College Dublin, Woodview House, Belfield, Dublin 4, Ireland; 2 Healthy Eating & Active Living Programme, Health Service Executive, Dublin, Ireland; 3 Department of Health, Dublin, Ireland

**Keywords:** Overweight, Obesity, Schoolchildren, Socioeconomic status, COSI

## Abstract

**Background:**

Existing evidence on the role of sociodemographic variables as risk factors for overweight and obesity in school-aged children is inconsistent. Furthermore, findings seem to be influenced by the obesity definition applied. Therefore, this study aimed to investigate if school sociodemographic indicators were associated with weight status in Irish primary schoolchildren and whether this association was sensitive to different obesity classification systems.

**Methods:**

A nationally representative cross-sectional sample of 7542 Irish children (53.9% girls), mean age 10.4 (±1.2SD) years, participating in the Childhood Obesity Surveillance Initiative in the 2010, 2012/2013 or 2015/2016 waves were included. Height, weight and waist circumference were objectively measured. Five definitions of obesity were employed using different approaches for either body mass index (BMI) or abdominal obesity. Associations between overweight and obesity and sociodemographic variables were investigated using adjusted multilevel logistic regression analyses.

**Results:**

Children attending disadvantaged schools were more likely to be overweight and obese than their peers attending non-disadvantaged schools, regardless of the obesity classification system used. Associations remained significant for the BMI-based obesity definitions when the sample was stratified by sex and age group, except for boys aged 8–10.5 years. Only boys aged ≥10.5 years in disadvantaged schools had higher odds of abdominal obesity (UK 1990 waist circumference growth charts: OR = 1.56, 95%CI = 1.09–2.24; waist-to-height ratio: OR = 1.78, 95%CI = 1.14–2.79) than those in non-disadvantaged schools. No associations were observed for school urbanisation level.

**Conclusions:**

School socioeconomic status was a strong determinant of overweight and obesity in Irish schoolchildren, and these associations were age- and sex-dependent. School location was not associated with overweight or obesity. There remains a need to intervene with school-aged children in disadvantaged schools, specifically among those approaching adolescence, to prevent a trajectory of obesity into adult life.

**Electronic supplementary material:**

The online version of this article (10.1186/s12889-018-5246-7) contains supplementary material, which is available to authorized users.

## Background

In 2013, 23.2% of children and adolescents in developed countries aged between 2 and 19 years were overweight or obese [1]. The second wave of the Childhood Obesity Surveillance Initiative (COSI) conducted in 2009/2010 showed that the prevalence of overweight and obesity, assessed according to the International Obesity Task Force (IOTF) cut-offs [2, 3], in European children aged between 6 and 9 years ranged from 10.8% to 45.1%, with the lowest rates observed in countries such as Belgium, Latvia and Lithuania, and the highest in Mediterranean countries such as Greece, Italy, Portugal or Spain [4]. According to the latest COSI wave conducted in the Republic of Ireland in 2015/2016, one in five children aged 6–12 years were overweight or obese [5]. Although Irish childhood obesity rates are not among the highest in Europe, Ireland is the country with the 8th highest childhood overweight and obesity prevalence in Europe [6]. In fact, it has been projected that by 2030 the prevalence of overweight and obesity in adults will reach levels of 89% and 85% in Irish males and females, respectively [7]. The increase in the prevalence of obesity in childhood and adolescence occurs in conjunction with the increase in the prevalence of other comorbidities including glucose intolerance, type 2 diabetes, hypertension, and hyperlipidaemia [8]. In addition, it is well known that overweight and obesity during childhood and adolescence track into adulthood and are associated with moderately increased risks of adult obesity-related morbidity, such as cardiovascular disease, cancer, and premature death [9].

Previous evidence acknowledges socioeconomic status (SES) as risk factor for obesity in young populations; however, studies are inconsistent in terms of the direction of this association. Findings from a recent meta-analysis showed that children aged 0–15 years belonging to lower SES groups were at 10% and 41% higher risk of overweight or obesity, respectively, than children in higher SES groups [10]. Findings from the Growing Up in Ireland project, a national longitudinal study of Irish children, showed no evidence of a difference in obesity prevalence across social classes in children aged 3 years old [11], whereas at 9 years of age, lower maternal education and lower household class were associated with higher odds of obesity [12]. This association still remains under-investigated among Irish school-aged children though. In the European region, a large epidemiological study of 11,994 2-to-9-year-old children observed a negative SES gradient in the prevalence of overweight and obesity in 5 out of the 8 participating countries and concluded that the association between SES factors and weight status was heterogeneous across different European regions [13]. There is a wide variety of SES indicators employed across studies that could explain the lack of agreement in the association between SES and weight status in children. Most of these indicators are based on individual or family measures such as family income, parental education level or parental employment status whereas the use of broader indicators at group or at setting level, i.e. school level, are not frequently used. The use of a global indicator could provide a more holistic estimate of the environment to which the child is exposed. Furthermore, few studies have addressed these associations separately by sex and/or age [13] and, for that reason, existing evidence to identify target groups for childhood obesity prevention is very limited.

Available evidence on the association between weight status and urban versus rural areas is also inconclusive. Several studies conducted in Europe have reported a higher prevalence of obesity in children living in rural areas in comparison with those living in urban areas [14–16]. In contrast, the Greek COSI study showed higher obesity rates among children living in cities [17] whereas other studies have failed to show differences in obesity prevalence between urban and rural areas [18]. In Ireland, no data are available on a potential rural vs urban gradient in terms of childhood obesity.

Body mass index (BMI) is the most widely accepted tool in epidemiological studies and clinical practice to diagnose excess body weight in both children and adults. In children, there is no a clear agreement on the BMI-based obesity classification approach that should be used to identify overweight and obesity. Due to the fact that each classification system is based on specific reference populations, the estimates of overweight and obesity prevalence differ based on the cut-offs applied [17, 19–21] and, therefore, the direction of the associations under evaluation. Furthermore, waist circumference (WC) and the waist-to-height ratio (WHeR) are considered good indicators of abdominal obesity and their use is becoming increasingly popular due to their association with cardiometabolic risk factors [22].

Given the long-term consequences of childhood obesity and the impact on adult health, research to elucidate the association between sociodemographic factors and the risk of overweight and obesity deserves more attention. Therefore, this study aimed to assess the association between school sociodemographic variables, i.e. schools’ SES and urbanisation level, and weight status, defined according to several obesity definitions, in 8- to 12-year-old Irish primary schoolchildren. We focused on this age group because this is the period that precedes puberty and, at these ages, the identification of obesity could predict the condition in adulthood [9, 23]; hence, identification of children at higher risk of obesity before the onset of puberty might be crucial to prevent excess weight gain during this period and during adulthood. Moreover, Ireland has been recognised to have significant levels of health inequalities, which appear to have worsened since the 2008 recession [24]. These findings will aid the Irish national Health authorities, whose aim is to reduce health and social inequalities, to identify populations groups at higher risk of overweight and obesity as potential targets for policy-making strategies.

## Methods

### Subjects and study design

The WHO European COSI is a collaborative study that was initiated in 2008 by the WHO Regional Office for Europe with 13 Member States (Belgium, Bulgaria, Cyprus, Czech Republic, Ireland, Italy, Latvia, Lithuania, Malta, Norway, Portugal, Slovenia and Sweden). Currently, COSI includes 35 European countries co-operating in relation to survey content, methodology and timing using a common European protocol [25]. The study aims to routinely measure overweight and obesity prevalence of primary schoolchildren to monitor the progress of the obesity epidemic in this population group, allow between-country comparisons within the WHO European Region and inform action to reverse the trend [4]. COSI is a unique system that provides a large dataset based on nationally representative samples and standardised weight and height measurements. A total of four rounds have been conducted to date. The COSI data collection rounds took place during the following school years: Round 1 in 2008, Round 2 in 2009/2010, Round 3 in 2012/2013, and Round 4 in 2015/2016 [25].

This study focuses on a cross-sectional sample of 7542 children (53.9% girls) aged 8–12 years (mean = 10.4 ± 1.2 years) attending primary schools in the Republic of Ireland in 2010, 2012/2013 or 2015/2016. Children measured in Round 1 were < 8 years and were excluded from this study. In wave 2, children aged 8–9 years old were examined between October and November 2010; measurements in Round 3 took place between November 2012 and January 2013 in children aged 8–11 years old, and 9–12-year-old children were measured in Round 4 between November 2015 and February 2016. Ethical approval was obtained from the Research Ethics Committee, Human Research Sub Committee, University College Dublin, on all occasions and all the study procedures were performed in accordance with the ethical standards laid down in the 1964 Declaration of Helsinki and its later amendments. Consent was obtained at school, parent and child level for each COSI round. An initial letter and a consent form were sent to the principals. Subsequently, all parents from the sampled classes with the selected age groups in participating schools were fully informed about all study procedures and a signed informed consent was obtained on a voluntary basis prior to the child’s enrolment to the study. On the day of the measurement, verbal consent from the child to participate in the study was obtained and the child’s response was registered on the examination record form. The Research Ethics Committee gave their approval to obtain verbal consent from the child to take part in the study.

Cluster sampling was applied with the school as primary sampling unit. Details about the cluster-sampling procedure and the sample size calculations have already been described elsewhere [25]. In summary, 163 schools consented to take part in the study in Round 1 (2008). Only one class per school with the target age group was randomly sampled, even if there were multiple classes in the school with the same age range. All children in the sampled class were invited to participate. Those same 163 schools were contacted again for Round 2, Round 3 and Round 4 for data collection in 2010, 2012/2013 and 2015/2016, respectively. Also for Rounds 2, 3 and 4, only one class from each year was selected per school. As classes were selected at random, the same classes might have been selected across rounds and children at older ages could have been measured more than once. In case a child had more than one measurement, only the first measurement was included in the present analysis.

### Physical examinations

All researchers attended a training session in anthropometric procedures and data collection. Anthropometric measurements were carried out following a standardised protocol drawn up by the WHO for weight, height and WC [26]. Children were asked to wear normal, light, indoor clothing without shoes. For Round 2 (2010), SECA 872 weighing scales and SECA 214 portable stadiometres were used throughout. For Round 3 (2012/2013) and Round 4 (2015/2016), Leicester Height Measure portable stadiometres were used. Weight was measured with HD-305 Tanita scales in Round 3 and with Tanita WB-100 MA scales in Round 4. For all rounds, weighing scales were calibrated prior to the start of the data collection. Weight was measured in kilograms, to the nearest 0.1 kg. Children’s height was measured in centimetres and the reading taken to the last completed 0.1 cm. BMI was calculated from the formula: weight (kg) divided by height squared (m^2^). WC was measured in cm at the midpoint between the lowest rib and the iliac crest and recorded to the nearest mm. In 2010, WC measurement was taken with a non-elastic metal tape with blank lead-in whereas a non-stretchable plastic tape with a clear plastic slider with cursor line was used in 2012/2013 and 2015/2016. Extreme values were checked and children with unrealistic WC measurements (< 30 cm or > 110 cm) compared to their weight and height were excluded (*n* = 2).

Three BMI-based definitions were used to assess overall obesity and two definitions were applied for abdominal obesity. The International Obesity Task Force (IOTF) [2, 3], the WHO 2007 [27] and the Centers for Disease Control and Prevention 2000 (CDC 2000) (≥85th percentile cut-off) [28, 29] age- and sex-specific BMI cut-off points were used to identify overweight including obesity. More specific details about these cut-off points are available in Additional file 1. Children were categorised into two weight status categories, that is, underweight/normal weight and overweight/obese, according to the three BMI-based definitions. Children with BMI ≥85th percentile according to the CDC 2000 cut-off points, with BMI ≥1 standard deviation above the mean using the WHO 2007 growth curves, and categorised as either 1 or 2 with the IOTF cut-offs were classified as overweight/obese. Abdominal obesity, including overweight, was defined as WHeR > 0.5 [22] and ≥91st percentile according to the United Kingdom 1990 (UK 1990) reference growth charts for waist [30].

### School characteristics

Data on school year, school name, school address and school location were collected through the school core data collection form. Schools were divided into ‘urban schools’ or ‘rural schools’ based on their location. An urban area was defined as having population clusters of ≥1500 inhabitants, and a rural area referred to areas < 1500 inhabitants [31]. Disadvantaged schools, defined as those schools at social or economic disadvantage, were identified by the Irish Department of Education and Skills [32]. The identification of disadvantaged schools was based on the following variables: unemployed parents, percentage of local authority accommodation, percentage of lone parenthood, percentage of Travellers, percentage of children eligible for free book grants and percentage of large families (i.e. ≥5 children) [33]. Thus, schools were split based on their SES level into ‘disadvantaged schools’, i.e. those at greater socioeconomic disadvantage, and ‘non-disadvantaged’.

### Other data

Individual information on date of birth, date and time of measurement and sex were obtained. The child’s age was calculated using the formula: (date of measurement - date of birth)/365.25.

### Data analysis

The statistical software package Stata version 13.0 (StataCorp LP) was used to perform the analyses. Distribution of all variables was checked before the analysis. Children were split into younger children (< 10.5 years) and older children (≥10.5 years) according to the median age of the sample. Characteristics of the study sample are presented as medians and percentiles for continuous variables and as percentages for categorical variables. Chi-squared tests were performed to compare the prevalence of overweight and obesity across sexes, age-, school SES and schools urbanisation groups within each obesity definition. The percent agreement between definitions and the kappa statistic were computed as a measure of agreement. Multilevel logistic regression analyses were performed to investigate the association between school characteristics (independent variables) and the prevalence of overweight and obesity (dependent variables) in this sample of Irish schoolchildren. Sex, age, height, school disadvantaged status, school location and measurement round were entered into the model. The variable school was entered as random intercept for the BMI-based definitions and as random slope for the abdominal obesity definitions. The threshold for statistical significance was set at *p* ≤ 0.05. *P*-values were corrected for multiple testing with the Benjamini-Hochberg False Discovery Rate [34].

## Results

### Prevalence of overweight and obesity according to BMI-based definitions

**Fig. 1:**
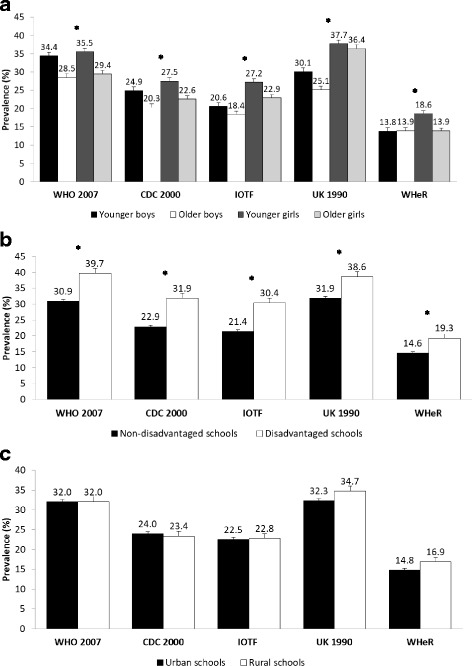
Prevalence (%) and 95% confidence intervals of overweight and obesity among Irish COSI children separately by sex and age group (**a**), school socioeconomic status (**b**), and the degree of school urbanisation (**c**). *Chi-squared test, within-group significant *p*-values after correction for multiple testing with the Benjamini- Hochberg False Discovery Rate [34]. CDC, Centers for Disease Control and Prevention; IOTF, International Obesity Task Force; UK, United Kingdom; WHeR, waist-to-height ratio; WHO, World Health Organisation. Younger children < 10.5 years; older children ≥10.5 years

Baseline characteristics of the sample are displayed in Table 1. The prevalence of overweight and obesity according to age and sex is shown in Fig. 1a. Focusing on BMI cut-off points, the prevalence of overweight and obesity was higher with the WHO 2007 definition (35.5%–28.5%) as compared with either the IOTF (27.2%–18.4%) or the CDC 2000 (27.5%–20.3%) cut-offs, which yielded similar estimates.

**Table 1:** Baseline characteristics of the Irish COSI study sample separately by sex

	All (*n* = 7542)		Boys (*n* = 3476)		Girls (*n* = 4066)	
	median	25th–75th	median	25th–75th	median	25th–75th
Age (years)	10.4	9.3–11.5	10.5	9.4–11.5	10.4	9.3–11.4
Weight (kg)	36.1	30.9–42.9	36.0	31.1–42.0	36.2	30.8–43.7
Height (cm)	142.4	135.9–149.5	142.9	136.5–149.4	141.9	135.4–149.5
WC (cm)	62.4	58.3–68.2	62.5	58.8–68.0	62.3	57.9–68.4
BMI (kg/m^2^)	17.6	16.2–19.8	17.5	16.1–19.3	17.8	16.2–20.2
	n	%	n	%	n	%
School socioeconomic status						
Non-disadvantaged schools	6636	88.0	3091	88.9	3545	87.2
Disadvantaged schools	906	12.0	385	11.1	521	12.8
School location						
Urban	6303	83.6	2888	83.1	3415	84.0
Rural	1239	16.4	588	16.9	651	16.0

*BMI* body mass index, *WC* waist circumference

### Prevalence of abdominal overweight and obesity

The UK 1990 cut-off points provided greater rates of abdominal obesity (37.7%–25.1%) than the WHeR estimates (18.6%–13.8%). Girls and younger children had higher overweight or obesity rates than boys and older children, respectively, across all obesity definitions (Fig. 1a). Overweight and obesity rates were significantly different (*p* < 0.001) across sexes and age-groups regardless of the obesity definition applied (see Additional file 2: Table S1). BMI-derived overweight and obesity and abdominal obesity rates were significantly (*p* < 0.001) higher among children attending disadvantaged schools than among those in non-disadvantaged schools (Fig. 1b). Urban and rural schools had similar rates of childhood overweight and obesity with all the obesity classification systems applied; no significant differences were observed (Fig. 1c).

### Comparison of overweight and obesity prevalence across different definitions

Overall prevalence of overweight or obesity significantly (*p* < 0.001) differed among the three BMI-based definitions (Table 2). A significant difference (*p* < 0.001) was also observed between the two abdominal obesity definitions. The highest discrepancies between definitions were observed for abdominal obesity definitions as 17.7% of the children were not classified to the same group. The kappa statistic showed a moderate agreement (κ = 0.53) between the two definitions in terms of classifying children to the same weight status group. A very good agreement (κ = 0.95) was found between the CDC 2000 and the IOTF cut-offs as the disagreement rate was very low (1.8%). The WHO 2007 cut-offs showed good agreement with both the CDC 2000 (κ = 0.80) and the IOTF (κ = 0.76) cut-offs, although the disagreement rate was lower with the CDC 2000 (8.2%) than with the IOTF (9.5%) cut-offs.

**Table 2:** Cross-classification analyses among obesity definitions in children participating in the Irish COSI study

	Same category (%)	Opposite category (%)	κ	*p*-value*
WHO 2007 vs CDC 2000	91.8	8.2	0.80	< 0.001
WHO 2007 vs IOTF	90.5	9.5	0.76	< 0.001
CDC 2000 vs IOTF	98.2	1.8	0.95	< 0.001
UK 1990 vs WHeR	82.3	17.7	0.53	< 0.001

*p < 0.05, chi-squared test

κ, kappa statistic

*BMI* body mass index, *CDC* Centers for Disease Control and Prevention, *IOTF* International Obesity Task Force, *UK* United Kingdom, *WHeR* waist-to-height ratio, *WHO* World Health Organisation

### Associations between school characteristics and overweight and obesity

The results of the multilevel logistic regression analyses are displayed in Table 3. Regardless of the obesity definition applied, i.e. abdominal obesity or BMI cut-offs, children attending disadvantaged schools were more likely to be overweight or obese than their peers attending non-disadvantaged schools (ranging from OR = 1.40, 95% CI = 1.10–1.77 to OR = 1.60, 95% CI = 1.30–1.96). When analyses were split by sex and age, we consistently observed across all BMI-based cut-offs that girls and older boys in disadvantaged schools had higher odds of being overweight and obese than those in non-disadvantaged schools; this association was not significant among younger boys. For abdominal obesity, only older boys in disadvantaged schools were more likely to be overweight or obese than those in non-disadvantaged schools (UK 1990: OR = 1.56, 95% CI = 1.09–2.24; WHeR, OR = 1.78, 95% CI = 1.14–2.79). No associations were observed between school urbanisation level and overweight or obesity for any of the definitions. No interactions were observed across covariates.

**Table 3:** Multilevel logistic regression analyses across obesity definitions among children participating in the Irish COSI study

	All children (n = 7542)			Boys (n = 3476)						Girls (n = 4066)					
				Younger boys (*n* = 1701)			Older boys (*n* = 1775)			Younger girls (*n* = 2080)			Older girls (*n* = 1986)		
	OR^a^	95% CI	*p*-value*	OR^a^	95% CI	*p*-value*	OR^a^	95% CI	*p*-value*	OR^a^	95% CI	*p*-value*	OR^a^	95% CI	*p*-value*
WHO 2007															
Sex (ref. boy)	1.04	0.93–1.15	0.515	–	–	–	–	–	–	–	–	–	–	–	–
Age (ref. young children)	0.83	0.79–0.91	**< 0.001**	–	–	–	–	–	–	–	–	–	–	–	–
School SES (ref. non-disadvantaged schools)	1.50	1.25–1.79	**< 0.001**	1.13	0.82–1.57	0.449	1.58	1.14–2.17	**0.005**	1.68	1.21–2.34	**0.002**	1.72	1.26–2.33	**0.001**
School urbanisation(ref. urban)	1.04	0.89–1.22	0.627	1.16	0.88–1.53	0.297	1.08	0.82–1.41	0.595	1.07	0.79–1.44	0.674	0.90	0.68–1.20	0.482
Measurement round	0.83	0.77–0.89	**< 0.001**	0.78	0.68–0.88	**< 0.001**	0.75	0.60–0.92	**0.006**	0.85	0.75–0.95	**0.004**	0.98	0.80–1.19	0.811
CDC 2000															
Sex (ref. boy)	1.14	1.01–1.28	0.032	–	–	–	–	–	–	–	–	–	–	–	–
Age (ref. young children)	0.85	0.75–0.95	**0.005**	–	–	–	–	–	–	–	–	–	–	–	–
School SES (ref. non-disadvantaged schools)	1.59	1.29–1.95	**< 0.001**	1.20	0.85–1.71	0.306	1.79	1.25–2.55	**0.001**	1.89	1.30–2.74	**0.001**	1.77	1.23–2.53	**0.002**
School urbanisation(ref. urban)	1.02	0.85–1.23	0.828	1.03	0.75–1.40	0.867	0.94	0.69–1.30	0.717	1.19	0.85–1.67	0.313	0.96	0.69–1.33	0.788
Measurement round	0.82	0.75–0.88	**< 0.001**	0.76	0.66–0.88	**< 0.001**	0.76	0.60–0.96	**0.020**	0.81	0.72–0.92	**0.001**	0.98	0.78–1.22	0.832
IOTF															
Sex (ref. boy)	1.37	1.21–1.54	**< 0.001**	–	–	–	–	–	–	–	–	–	–	–	–
Age (ref. young children)	0.89	0.79–1.00	0.054	–	–	–	–	–	–	–	–	–	–	–	–
School SES (ref. non-disadvantaged schools)	1.60	1.30–1.96	**< 0.001**	1.15	0.80–1.65	0.457	1.73	1.22–2.47	**0.002**	1.91	1.31–2.77	**0.001**	1.83	1.29–2.60	**0.001**
School urbanisation(ref. urban)	1.08	0.90–1.30	0.412	1.22	0.89–1.66	0.217	1.02	0.74–1.40	0.913	1.21	0.86–1.69	0.277	0.96	0.69–1.33	0.789
Measurement round	0.84	0.77–0.92	**< 0.001**	0.77	0.67–0.89	**0.001**	0.80	0.63–1.02	0.075	0.85	0.75–0.97	**0.012**	0.98	0.79–1.22	0.853
UK 1990															
Sex (ref. boy)	1.60	1.42–1.79	**< 0.001**	–	–	–	–	–	–	–	–	–	–	–	–
Age (ref. young children)	0.30	0.26–0.35	**< 0.001**	–	–	–	–	–	–	–	–	–	–	–	–
Height (cm)	1.11	1.10–1.12	**< 0.001**	1.15	1.12–1.17	**< 0.001**	1.09	1.08–1.11	**< 0.001**	1.14	1.12–1.16	**< 0.001**	1.09	1.08–1.11	**< 0.001**
School SES (ref. non-disadvantaged schools)	1.40	1.10–1.77	**0.006**	1.35	0.95–1.92	0.095	1.56	1.09–2.24	**0.016**	1.61	1.04–2.50	0.032	1.44	0.96–2.14	0.075
School urbanisation(ref. urban)	1.12	0.91–1.38	0.276	1.39	1.03–1.88	0.030	1.06	0.78–1.43	0.715	1.04	0.71–1.52	0.848	1.16	0.82–1.62	0.397
Measurement round	0.58	0.54–0.63	**< 0.001**	0.52	0.45–0.61	**< 0.001**	0.58	0.45–0.74	**< 0.001**	0.56	0.49–0.64	**< 0.001**	0.61	0.49–0.77	**< 0.001**
WHeR															
Sex (ref. boy)	1.17	1.02–1.36	0.031	–	–	–	–	–	–	–	–	–	–	–	–
Age (ref. young children)	0.92	0.80–1.06	0.237	–	–	–	–	–	–	–	–	–	–	–	–
School SES (ref. non-disadvantaged schools)	1.46	1.12–1.89	**0.005**	1.40	0.93–2.10	0.108	1.78	1.14–2.79	**0.011**	1.52	0.98–2.34	0.059	1.46	0.94–2.27	0.093
School urbanisation(ref. urban)	1.23	0.98–1.54	0.078	1.27	0.87–0.98	0.191	1.05	0.71–1.55	0.817	1.22	0.83–1.80	0.306	1.43	0.98–2.09	0.068
Measurement round	0.79	0.98–1.54	**< 0.001**	0.82	0.69–0.98	0.025	0.77	0.58–1.01	0.063	0.75	0.65–0.87	**< 0.001**	0.79	0.61–1.04	0.092

^a^Adjusted for random effects at school level

^*^p-values in bold font are significant after correcting for multiple testing with the Benjamini- Hochberg False Discovery Rate [34]

Younger children < 10.5 years; older children ≥10.5 years

*CDC* Centers for Disease Control and Prevention, *CI* confidence interval, *IOTF* International Obesity Task Force, *OR* odds ratio, *SES* socioeconomic status, *UK* United Kingdom, *WHeR* waist-to-height ratio, *WHO* World Health Organisation

## Discussion

Overall, our findings suggest that children attending disadvantaged schools are at higher risk of being overweight or obese than children in non-disadvantaged schools, specifically girls and boys older than 10.5 years. Furthermore, older boys in disadvantaged schools were more likely to be abdominally obese as compared with boys in non-disadvantaged schools. The present study examined the association between schools’ SES and urbanisation level and overweight and obesity, applying a range of obesity cut-offs, in a large representative sample of 8- to 12-year-old Irish schoolchildren. To the best of our knowledge, this is the first study that evaluated the association between school sociodemographic characteristics and overweight or obesity in schoolchildren separately for boys and girls and age groups.

Our results showed that the prevalence of overweight and obesity in this sample of primary schoolchildren largely differed across obesity definitions. Overall, the highest prevalence was yielded by the UK 1990 definition and the lowest by the WHeR, both being measurements of abdominal obesity. The UK 1990 growth charts were developed nearly 30 years ago [30] before the worldwide shift towards more overweight and obese populations, including children, had occurred. Therefore, the UK 1990 growth charts could be more indicative of what a normal population is since the more recent growth charts could be distorted by the current greater rates of overweight and obesity. In contrast, the use of the WHeR has become popular in the past years for its direct association with cardiometabolic risk factors and for being considered as good marker of both total and trunk adiposity in children and adolescents [35]. We observed that the level of disagreement between these two definitions was about 20%, which can be considered relatively high. However, this lack of agreement between the two definitions was mostly due to those children that were borderline to be classified as abdominally obese by either one or the other definition. Only 8 children labelled as abdominally obese by the WHeR were below the 91st percentile of the 1990 UK growth charts and 3% (*n* = 203) of the children classified as overweight or obese by the UK 1990 cut-offs were below a WHeR of 0.45. The 1990 UK growth charts seemed to be more sensitive as they labelled children as abdominally obese when their WHeR was between 0.45 and < 0.5, but who did not reach the 0.5 threshold. While it is not our intention to make a recommendation on the most adequate measure that should be employed, the definition that allows a better identification of those children with overweight or obesity and/or at greater risk of developing related chronic disease later in life should be preferred.

Focusing on the BMI definitions, our findings were consistent with previous studies that showed the WHO and the IOTF criteria yielding the highest and lowest prevalence estimates, respectively [17, 20, 21]. The CDC and the IOTF cut-offs provided very similar overweight and obesity rates and showed a very good level of agreement whereas the highest disagreement rates were observed with the WHO growth charts. Differences in prevalence estimation can be expected given that these definitions were developed with different objectives and sources of reference populations [2, 3, 27, 28]. While the CDC and the IOTF cut-offs are a description of reference populations, the WHO growth curves represent a desired standard [21]. Like the UK 1990 growth charts, the WHO reference data was based on BMI data collected before the obesity epidemic [21], which could also explain the higher overweight and obesity rates yielded by this definition. In surveillance, the selection of the classification system to define overweight and obesity in children is a critical step; it will have implications regarding international and between-studies comparisons, describe trends over time and draw conclusions for policy development purposes.

This study applied five obesity classification systems to investigate the association between school sociodemographic characteristics and weight status in this sample of Irish schoolchildren. Despite the discrepancies in prevalence estimation across definitions, our results consistently showed that children attending disadvantaged schools were at greater risk of being overweight or obese than their peers attending non-disadvantaged schools regardless of the classification system applied. In line with our results, a meta-analysis conducted by Wu et al. [10] concluded that children aged 0–15 years from lower socioeconomic groups were more likely to be overweight or obese. Although the authors stated that the use of different definitions of overweight and obesity applied across studies was a study limitation, our findings showed that the inverse association between SES and weight status was independent of the classification system used. Likewise in Ireland, Keane et al. [12] observed that 9-year-old children from lower household class families and those with lower educated parents had higher odds of being obese. However, no differences in obesity rates across social classes were observed when these children were aged 3 years [11]. A social pattern for obesity was reported in Irish adults with higher percentages of obesity among those from lower social classes; no social pattern was observed for overweight though [36]. In our study, an indicator of school SES was applied rather than an indicator of household SES. The measure provides an indicator of the socioeconomic characteristics of the community surrounding the school and of the environment to which the child is permanently exposed.

Focusing on subgroup analysis, Wu et al. [10] showed that boys, but not girls, with low SES were at higher risk of overweight or obesity; however, according to the authors, the number of studies addressing this association in boys and girls separately is not sufficient yet to draw more solid conclusions. Interestingly, our findings showed that the association was significant in girls and in boys in the older group when the BMI-based definitions were applied, but not among younger children. In contrast to the results in the meta-analysis [10], only younger girls attending disadvantaged schools were at higher risk of overweight or obesity. These findings suggest that the odds of being overweight or obese is higher in girls in disadvantaged schools than in those attending non-disadvantaged schools independently of their age whereas boys in disadvantaged schools are at risk of overweight or obesity when they become older but not at younger ages. Because WC and WHeR are considered good markers of adiposity-related morbidities and are strongly associated with cardiometabolic risk factors [22], boys aged 10.5 years and older in disadvantaged schools could represent a population group at higher risk of future chronic diseases such as cardiovascular diseases. However, we could not investigate other factors that might explain the potential role of sex and age in this association. Overall, and compared with their peers in non-disadvantaged schools, these children may be more exposed to an obesogenic environment that promotes weight gain and obesity. The specific characteristics of deprived communities together with low family SES are likely to lead these children to diets rich in low-cost energy-dense food and reduced opportunities to engage in sports and active play [10]. Therefore, children attending disadvantaged schools, mainly girls and boys older than 10.5 years, deserve special attention and should be one of the targets of public health policies aiming to prevent obesity in Ireland.

We failed to observe significant associations between school location, i.e. urban vs rural, and weight status in this sample of Irish school-aged children. Likewise, the Irish National Health and Lifestyle Surveys (SLÁN) conducted in 2002 reported little or no difference in levels of obesity between those adults living in rural or urban areas [37]. In Europe, Hassapidou et al. [17] observed differences in abdominal obesity prevalence in school-aged Greek children living in rural or urban areas whereas no differences were found when obesity was defined according to BMI. They showed higher prevalence of abdominal obesity in children living in the capital, Athens, than in those living in villages and small cities [17]. Likewise, Rush et al. [38] observed in a large sample of 5- and 10-year-old New Zealand children that those living in an urban setting had higher rates of overweight and obesity than those living in a rural area. On the other hand, evidence from other studies conducted in Europe showed that the prevalence of both BMI-defined obesity and abdominal obesity was lower in children living in cities as compared with those living in rural areas [14–16]. The lack of agreement among studies could be explained by country-specific urban-rural discrepancies in overweight and obesity-related factors such as the lifestyle and/or socioeconomic indicators. Differences in the definition of rural-urban locations across countries could be another reason explaining the lack of agreement among studies. Nevertheless, the association between school location and obesity should be investigated more in depth to confirm previous findings and to identify a potential obesity gradient according to urbanisation level. To date, this link seems to be country-specific.

### Strengths & limitations

The COSI Irish study represents a large and nationally representative sample of Irish schoolchildren aged 8 to 12 years old. Objective measurements on anthropometric variables were taken by trained nutritionists following a standardised protocol. Another strength is that measurements were collected following a standardised surveillance methodology. Strict adherence to the original protocol was reached and, as a result, the collected data will be integrated into the unique international COSI European database on overweight and obesity to perform multiple intercountry comparisons. Since the examinations across rounds took place during the same period of the year, i.e. autumn and winter, any potential seasonal effects were removed. In addition, information on schools’ SES was provided by the Irish Department of Education and Skills; therefore, answers are not subject to response bias. Furthermore, differences in the performance of obesity definitions that could influence the observed results can be discarded.

The study is also subject to some limitations. The analyses were performed without applying sampling weights to adjust for the sampling design, oversampling and non-response rates, which limits the generalization of the results to the entire population. The relatively low participation rate (63%) is another study limitation and, therefore, we cannot rule out a certain degree of response bias. Nevertheless, it should be noted that participation rates remained similar across rounds and classes and that the participating schools were representative of all primary schools in Ireland [39]. Furthermore, the cross-sectional nature of these analyses provides a transversal perspective of schools’ SES and urbanisation level as correlates of overweight and obesity in schoolchildren and cannot be used to establish causal relationships. The fact that we used a school-based SES indicator could be seen as a factor limiting comparability with other studies. Besides, misclassification bias cannot be ruled out as there might be children attending low SES schools that belong to higher SES families, and viceversa, although this figure would be very small. Another limitation is the use of different measuring equipment across waves as certain degree of measurement error cannot be precluded. However, data quality procedures were meticulously monitored throughout each measurement period to minimise this effect. It should be kept in mind that other factors such as parental nutrition knowledge, family feeding habits and/or family structure, amongst others, could have influenced the observed results. The COSI protocol includes a parentally reported questionnaire that captures some of these factors; however, these data were not available for all the children included in these analyses [40].

## Conclusions

In conclusion, school SES emerged as a crucial determinant of overweight and obesity in Irish schoolchildren, whereas no associations were observed with school location. Furthermore, we showed that these associations may be dependent of age and sex, which is the novelty of the study. Girls in disadvantaged schools, regardless of their age, were at higher risk of being overweight or obese whereas boys older than 10.5 years in these schools were more likely to be both overall overweight or obese and abdominally obese. From a public health perspective, these findings suggest that age and sex should be considered to develop more targeted strategies adapted to the socioeconomic dimension of the population. Children attending disadvantaged schools deserve special attention, especially as they approach adolescence, and health promotion policies should target the obesogenic environment they are exposed to. Associations with school location were not confirmed; therefore, more research is needed to shed light into the specific potential role that the degree of school urbanisation plays on the development of overweight and obesity in children. Overall, our findings provide more insights into the aetiology of childhood overweight and obesity not only in Ireland, but also in Europe, and will inform the development of tailored interventions and prevention programmes targeted to children in the European region and even beyond. More studies are needed, mainly with a longitudinal design, addressing these associations in other young populations and exploring other SES indicators.

## Additional files

Additional file 1: Overweight and obesity definitions. Detailed information is provided on the IOTF, CDC 2000 and WHO 2007 cut-offs used in this study to define overweight and obesity school-aged children. (DOCX 17 kb)

Additional file 2: Prevalence of overweight and obesity according to different definitions among Irish children COSI study. Percentages, 95% confidence intervals and Chi-squared *p*-values are provided to show the prevalence of overweight and obesity in school-aged children by age and sex, school socioeconomic level and school urbanisation level using three body mass index-based definitions and two abdominal obesity definitions. (DOCX 18 kb)

## Abbreviations

BMI: Body mass index
CDC: Centers for Disease Control and Prevention
COSI: Childhood Obesity Surveillance Initiative
IOTF: International Obesity Task Force
SES: Socioeconomic status
UK: United Kingdom
WC: Waist circumference
WHeR: Waist-to-height ratio
WHO: World Health Organisation
