# Supplementary material for: School sociodemographic characteristics and obesity in schoolchildren: does the obesity definition matter?
Source: BMC Public Health. 2018 Mar 9;18:337. doi: 10.1186/s12889-018-5246-7 (PMC5845160; doi:10.1186/s12889-018-5246-7)
Supplement: Supplementary file 1 — Overweight and obesity definitions. Detailed information is provided on the IOTF, CDC 2000 and WHO 2007 cut-offs used in this study to define overweight and obesity school-aged children. (DOCX 17 kb) [file 12889_2018_5246_MOESM1_ESM.docx]

**Additional file 1**

*The International Obesity Task Force (IOTF) cut-off points*

The IOTF cut-offs were developed in 2000 to create an internationally acceptable age- and sex- dependent definition of child overweight and obesity based on body mass index (BMI) measurements. Data from nearly 200,000 children and adolescents aged 0-25 years old were collected through six large nationally representative cross-sectional growth studies conducted in Brazil, Great Britain, Hong Kong, the Netherlands, Singapore, and the United States. The main purpose was to extrapolate the adult cut-off points of 25 and 30 kg/m^2^ to specify age- and sex-specific cut-off points for children and adolescents. Therefore, centile curves were generated for each of the surveys so that at age 18 years the curves passed through the BMI cut-off points of 25 and 30 kg/m^2^ for adult overweight and obesity. The resulting curves were averaged to provide age- and sex-specific cut-off points from 2 to 18 years. The definition is considered less arbitrary and more international than others and it is encouraged for direct comparison of trends in child obesity worldwide (Cole et al. BMJ. 2000; 320:1240).

*The Centers for Disease Control and Prevention (CDC) cut-off points*

The CDC created in 2000 the CDC Growth charts for the United States (US). The cut-off points are based on the growth curves that were developed with the data collected in five US cross-sectional nationally representative health examination surveys: NHES II (1963–65) and III (1966–70), and NHANES I (1971–74) II (1976–80), and III (1988–94). In 2007, it was recommended that, based on these growth curves, children with a BMI at or above the 95^th^ percentiless for age and sex should be considered obese and those with a BMI at or above 85^th^ but below 95^th^ should be considered overweigth (Kuczmarski RJ et al. Vital and health statistics Series 11, 2002(246):1-190; Krebs NF et al. Pediatrics. 2007; 120 Suppl 4:S193-228).

*The World Health Organization (WHO) cut-off points*

The WHO growth references for children aged 5 to 19 years were developed in 2007 according to the WHO Child Growth Standards for children under 5 years produced in 2006 and the widely used BMI cut-off points for adults. Data from the 1977 National Center for Health Statistics/WHO growth reference for children and adolescents aged 1-24 years were combined with data from the 0-5 years WHO Growth Standards to smooth the transition between the two datasets. Based on these growth curves, the WHO recommended that children with BMI of more than 2 standard deviations (+2 SD) above the mean should be considered obese and those with BMI between 1 (+1 SD) and +2 SD above the mean are overweigth. Two SD above the mean approximates the 97.7^th^ percentile and one SD to the 84^th^ percentile. At 19 years, the BMI values at +1 SD are 25.4 kg/m² for boys and 25.0 kg/m² for girls and the +2 SD value is 29.7 kg/m² for both sexes, aproximately. These values are equivalent to the overweight (>25.0 kg/m²) and obesity (>30.0 kg/m²) cut-offs points for adults (de Onis M et al. Bull World Health Organ. 2007; 85(9):660-667; Shields M et al. Int J Pediatr Obes. 2010; 5(3):265-273).
