# Supplementary material for: School sociodemographic characteristics and obesity in schoolchildren: does the obesity definition matter?
Source: BMC Public Health. 2018 Mar 9;18:337. doi: 10.1186/s12889-018-5246-7 (PMC5845160; doi:10.1186/s12889-018-5246-7)
Supplement: Supplementary file 2 — Prevalence of overweight and obesity according to different definitions among Irish children COSI study. Percentages, 95% confidence intervals and Chi-squared p-values are provided to show the prevalence of overweight and obesity in school-aged children by age and sex, school socioeconomic level and school urbanisation level using three body mass index-based definitions and two abdominal obesity definitions. (DOCX 18 kb) [file 12889_2018_5246_MOESM2_ESM.docx]

**Supplementary file 2**

**Table S1.** Prevalence of overweight and obesity according to different definitions among Irish children COSI study.

|  | **WHO 2007** | | | **CDC 2000** | | | **IOTF** | | | **UK 1990** | | | **WHeR** | | |
| --- | --- | --- | --- | --- | --- | --- | --- | --- | --- | --- | --- | --- | --- | --- | --- |
|  | **Prevalence** | | **p-value*** | **Prevalence** | | **p-value*** | **Prevalence** | | **p-value*** | **Prevalence** | | **p-value*** | **Prevalence** | | **p-value*** |
|  | **%** | **95% CI** |  | **%** | **95% CI** |  | **%** | **95% CI** |  | **%** | **95% CI** |  | **%** | **95% CI** |  |
|  |  |  |  |  |  |  |  |  |  |  |  |  |  |  |  |
| Younger boys | 34.4 | 32.2-36.7 | **<0.001** | 24.9 | 22.9-27.0 | **<0.001** | 20.6 | 18.8-22.6 | **<0.001** | 30.1 | 28.0-32.3 | **<0.001** | 13.8 | 12.2-15.5 | **<0.001** |
| Older boys | 28.5 | 26.5-30.6 |  | 20.3 | 18.5-22.3 |  | 18.4 | 16.7-20.3 |  | 25.1 | 23.2-27.2 |  | 13.9 | 12.3-15.6 |  |
| Younger girls | 35.5 | 33.5-37.6 |  | 27.5 | 25.6-29.4 |  | 27.2 | 25.3-29.1 |  | 37.7 | 35.7-39.9 |  | 18.6 | 16.9-20.3 |  |
| Older girls | 29.4 | 27.4-31.4 |  | 22.6 | 20.8-24.5 |  | 22.9 | 21.1-24.8 |  | 36.4 | 34.3-38.5 |  | 13.9 | 12.5-15.5 |  |
|  |  |  |  |  |  |  |  |  |  |  |  |  |  |  |  |
| Non-disadvantaged schools | 30.9 | 29.8-32.1 | **<0.001** | 22.9 | 21.9-23.9 | **<0.001** | 21.4 | 20.5-22.5 | **<0.001** | 31.9 | 30.8-33.0 | **<0.001** | 14.6 | 13.8-15.5 | **<0.001** |
| Disadvantaged schools | 39.7 | 36.6-43.0 |  | 31.9 | 28.9-35.0 |  | 30.4 | 27.4-33.4 |  | 38.6 | 35.5-41.9 |  | 19.3 | 16.9-22.0 |  |
|  |  |  |  |  |  |  |  |  |  |  |  |  |  |  |  |
| Urban schools | 32.0 | 30.9-33.2 | 0.987 | 24.0 | 23.0-25.1 | 0.635 | 22.5 | 21.4-23.5 | 0.763 | 32.3 | 31.2-33.5 | 0.097 | 14.8 | 14.0-15.7 | 0.064 |
| Rural schools | 32.0 | 29.4-34.6 |  | 23.4 | 21.1-25.9 |  | 22.8 | 20.6-25.3 |  | 34.7 | 32.1-37.4 |  | 16.9 | 14.9-19.0 |  |

*Chi-squared test. P-values in bold font are significant after correcting for multiple testing using the Benjamini- Hochberg False Discovery Rate.

CDC, Centers for Disease Control and Prevention; CI, confidence interval; IOTF, International Obesity Task Force; OR, odds ratio; UK, United Kingdom; WHeR, waist-to-height ratio; WHO, World Health Organisation.
